# Empirical cefepime+vancomycin versus ceftazidime+vancomycin versus meropenem+vancomycin in the treatment of healthcare-associated meningitis: results of the multicenter Ephesus study

**DOI:** 10.1186/s12879-023-08596-z

**Published:** 2023-09-28

**Authors:** Oguz Resat Sipahi, Deniz Akyol, Bahar Ormen, Gonul Cicek-Senturk, Sinan Mermer, Ugur Onal, Fatma Amer, Maysaa Abdallah Saed, Kevser Ozdemir, Elif Tukenmez-Tigen, Nefise Oztoprak, Ummugulsum Altin, Behice Kurtaran, Corneliu Petru Popescu, Mustafa Sakci, Bedia Mutay Suntur, Vikas Gautam, Megha Sharma, Safak Kaya, Eren Fatma Akcil, Selcuk Kaya, Tuba Turunc, Pınar Ergen, Ozlem Kandemir, Salih Cesur, Selin Bardak-Ozcem, Erkin Ozgiray, Taskın Yurtseven, Huseyin Aytac Erdem, Hilal Sipahi, Bilgin Arda, Hüsnü Pullukcu, Meltem Tasbakan, Tansu Yamazhan, Sohret Aydemir, Sercan Ulusoy

**Affiliations:** 1 Department of Infectious Diseases and Clinical Microbiology, Faculty of Medicine, Ege University, Izmir, Turkey; 2 Department of Infectious Diseases, Bahrain Oncology Center, King Hamad University Hospital, Muharraq, Bahrain; 3 Infectious Diseases Clinic, Kagizman State Hospital, Kagizman, Kars, Turkey; 4 Infectious Diseases and Clinical Microbiology Clinic, Izmir Katip Celebi University Ataturk Training and Research Hospital, Izmir, Turkey; 5 Infectious Diseases and Clinical Microbiology Clinic, SB Diskapi Yildirim Beyazit Training and Research Hospital, Ankara, Turkey; 6 Department of Infectious Diseases and Clinical Microbiology, Faculty of Medicine, Ekonomi University, Izmir, Turkey; 7 Department of Infectious Diseases and Clinical Microbiology, Faculty of Medicine, Uludag University, Bursa, Turkey; 8 Zagazig University, Zagazig, Egypt; 9 Department of Infectious Diseases and Clinical Microbiology, Pamukkale University, Denizli, Turkey; 10 Department of Infectious Diseases and Clinical Microbiology, Marmara University, Istanbul, Turkey; 11 Infectious Diseases and Clinical Microbiology Clinic, Antalya Education and Research Hospital, Antalya, Turkey; 12 Infectious Diseases and Clinical Microbiology Clinic, Bozyaka Training and Research Hospital, Izmir, Turkey; 13 Department of Infectious Diseases and Clinical Microbiology, Cukurova University, Adana, Turkey; 14 Dr Victor Babes Clinical Hospital of Infectious and Tropical Diseases, Bucharest, Romania; 15 Medical Student, Faculty of Medicine, Ege University, Izmir, Turkey; 16 Infectious Diseases and Clinical Microbiology Clinic, Adana Numune Training and Research Hospital, Adana, Turkey; 17 Post-Graduate Institute of Medical Education & Research, Chandigarh, India; 18 Department of Microbiology, All India Institute of Medical Sciences (AIIMS), Bilaspur, Himachal Pradesh India; 19 Infectious Diseases and Clinical Microbiology Clinic, Gazi Yasargil Education and Research Hospital, Diyarbakir, Turkey; 20 Department of Anaesthesiology and Reanimation, Istanbul University, Istanbul, Turkey; 21 Department of Infectious Diseases and Clinical Microbiology, Medical Faculty, Karadeniz Technical University, Trabzon, Turkey; 22 Department of Infectious Diseases and Clinical Microbiology, Baskent University, Adana, Turkey; 23 Department of Infectious Diseases and Clinical Microbiology, Istanbul Medeniyet University, Goztepe Educational and Research Hospital, Istanbul, Turkey; 24 Department of Infectious Diseases and Clinical Microbiology, Mersin University School of Medicine, Mersin, Turkey; 25 Infectious Diseases and Clinical Microbiology Clinic, Ankara Training Hospital, Ankara, Turkey; 26 Department of Infectious Diseases and Clinical Microbiology, Dr. Burhan Nalbantoğlu State Hospital, Nicosia, Northern Cyprus; 27 Department of Neurosurgery, Faculty of Medicine, Ege University, Izmir, Turkey; 28 Bornova Public Health Center, Izmir, Turkey; 29 Department of Clinical Microbiology, Faculty of Medicine, Ege University, Izmir, Turkey

**Keywords:** Healthcare-associated meningitis, Empirical therapy, Multicenter study, Glycopeptides, Antibiotics

## Abstract

**Background:**

Herein, we analyzed the efficacy of main antibiotic therapy regimens in the treatment of healthcare-associated meningitis (HCAM).

**Materials/methods:**

This retrospective cohort study was conducted in 18 tertiary-care academic hospitals Turkey, India, Egypt and Romania. We extracted data and outcomes of all patients with post-neurosurgical meningitis cases fulfilling the study inclusion criteria and treated with empirical therapy between December 2006-September 2018.

**Results:**

Twenty patients in the cefepime + vancomycin-(CV) group, 31 patients in the ceftazidime + vancomycin-(CFV) group, and 119 patients in the meropenem + vancomycin-(MV) group met the inclusion criteria. The MV subgroup had a significantly higher mean Glasgow Coma Score, a higher rate of admission to the intensive care unit within the previous month, and a higher rate of antibiot herapy within the previous month before the meningitis episode (*p* < 0.05). Microbiological success on Day 3–5, end of treatment (EOT) clinical success (80% vs. 54.8%% vs 57.9%), and overall success (EOT success followed by one-month survival without relapse or reinfection 65% vs. 51.6% vs. 45.3%), EOT all cause mortality (ACM) and day 30 ACM (15% vs. 22.6% vs. 26%) did not differ significantly (*p* > 0.05) among the three cohorts. No regimen was effective against carbapenem-resistant bacteria, and vancomycin resulted in an EOT clinical success rate of 60.6% in the methicillin-resistant staphylococci or ampicillin-resistant enterococci subgroup (*n* = 34).

**Conclusions:**

Our study showed no significant difference in terms of clinical success and mortality among the three treatment options. All regimens were ineffective against carbapenem-resistant bacteria. Vancomycin was unsuccessful in approximately 40% of cases involving methicillin-resistant staphylococci or ampicillin-resistant enterococci.

## Introduction

Despite advances in medicine, neurosurgery, antimicrobial therapy, and critical care, meningitis, specifically nosocomial or healthcare-associated meningitis (HCAM), still poses a significant risk of mortality and morbidity [1–3]. Prompt and appropriate treatment is essential in managing HCAM, as inappropriate treatment may lead to undesirable outcomes. In contrast to community-acquired meningitis (CAM), which is mainly caused by pneumococci and meningococci [4], healthcare-associated meningitis (HCAM) is typically caused by Gram-negative bacilli, such as *Pseudomonas aeruginosa*, *Acinetobacter* spp., or *Enterobacteriaceae*, and Gram-positive cocci, such as staphylococci or enterococci [1–3, 5–9]. This difference also results in a discrepancy in empirical antibiotic therapy. While the third-generation cephalosporin ± ampicillin and vancomycin or rifampin is recommended for empirical CAM therapy, HCAM empirical treatment is recommended to be a combination of vancomycin with ceftazidime or cefepime or meropenem [1, 2, 5, 7]. However, to our knowledge, there are no published clinical data comparing these empirical treatment regimens in terms of efficacy in HCAM. In this multicenter multinational retrospective cohort study, our main objective was to compare the microbiological and clinical efficacy of the main empirical antibiotic regimens used for HCAM.

## Methods

This study was conducted in 15 tertiary-care educational hospitals across nine cities in Turkey, including Izmir, Ankara, İstanbul, Adana, Denizli, Antalya, Diyarbakir, Trabzon, and Mersin, as well as three centers in India, Egypt, and Romania. We extracted data and outcomes from all adult patients (aged 18 or older) with post-neurosurgical meningitis who met the inclusion criteria and were treated with one of the three empirical therapy options. The study period was between December 2006 and September 2018. Demographic, clinical, and laboratory findings (white blood cells, CRP and other findings were on the day empirical antibiotic was started), predisposing factors, as well as information on treatment response and outcomes were obtained retrospectively.

### Inclusion criteria were as follows [9, 10]

- Age ≥ 18–year-old
- Presence of at least three of the following four clinical/laboratory criteria as meningitis findings:
  i) Cerebrospinal fluid (CSF) finding: ≥ 250 leucocytes/mm^3^
  ii) CSF culture positivity (in case of coagulase-negative staphylococci meningitis or culture negative meningitis ≥ 250/mm^3^ leucocytes was a necessity)
  iii) Body temperature > 38° C
  iv) At least one of the following clinical findings:; disturbances in consciousness, neck stiffness, nausea or vomiting.
- In case of a neurosurgical operation (except shunt operations) onset of the meningitis findings after at least 72 h of hospitalization and/or meningitis episode during the 30-day period after the neurosurgery and/or meningitis episode during the 90-day period after the shunt operations

### Exclusion criteria were as follows

- Age < 18-year-old
- In case the patient had > 1 meningitis episodes, only the first episode was included in the study.
- Meningitis episodes not fulfilling the above criteria.

Cefepime, ceftazidime, and meropenem were administered at a dosage of 2 g every 8 h, while vancomycin was given at a dosage of 500 mg every 6 h.

Cerebrospinal fluid (CSF) samples were obtained via lumbar puncture, percutaneous aspiration of the shunt reservoir, or puncture of the extra ventricular drainage tubing. The samples were routinely centrifuged, and the pellet was Gram-stained. Identification and determination of antimicrobial susceptibility were performed using the VITEK 2 automated system (BioMerieux Inc, Mercy L’etoil, France) or conventional methods. Antibacterial susceptibility tests were evaluated according to the Clinical Laboratory Standards Institute (CLSI) criteria until 2014 and the European Committee on Antimicrobial Susceptibility Testing (EUCAST) after 2015 [11, 12].

### Clinical and microbiological success criteria were as follows

#### Day 3–5 clinical response

Defervescence of fever and improvement of other clinical signs on days 3–5.

#### Day 3–5 microbiological response

Negative CSF culture with a decrease in leukocyte count on days 3–5.

#### End of treatment (EOT) clinical success

Absence of any need for modification of empirical treatment, along with concomitant clinical (i.e., defervescence of fever and improvement of other clinical signs) and Tmicrobiological response at the end of the empirical therapy regimen. Discontinuation of one of the combined antibiotics after receiving culture results was not considered a modification, but rather an indication of successful treatment.

#### All cause mortality (ACM)

This referred to death occurring during the meningitis episode, regardless of the cause.

#### Day 30 all cause mortality

ACM after 30-days after the start of the empirical meningitis treatment.

#### Infection related mortality

Mortality due to documented or clinically diagnosed infection/meningitis according to the oonsulting physician.

#### Overall clinical success

This referred to survival at the EOT with clinical and microbiological success, and no relapse or recurrence during the 30-day follow-up period.

### Statistical analysis

The aim of this study was to compare the clinical success, mortality, and overall clinical success rates for cohorts that received the three empirical regimens. We used SPSS version 25.0 (Chicago, IL, USA) for all analyses. We evaluated the difference between groups using the χ2 test and one-way ANOVA test, as indicated. The significance level was set at *p* < 0.05.

## Results

A total of 170 postneurological meningitis cases were included in the study, with 20 patients in the cefepime + vancomycin (CV) group, 31 in the ceftazidime + vancomycin (CFV) group, and 119 in the meropenem + vancomycin (MV) group. Of these patients, 28 (16.5%) had shunt infections, 20 (11.8%) were on lumbar drainage, and 36 (21.2%) were on external ventricular drainage at the time of diagnosis of nosocomial meningitis. Table 1 shows the underlying diseases, age, and other characteristics of the cases. There were no significant differences between the three therapy cohorts in terms of underlying diseases, shunt, lumbar or extraventricular drainage, treatment duration, gender, and age. However, admission to the intensive care unit (ICU) and any antibiotic therapy during the one-month prior to the meningitis episode were significantly more common in the MV subgroup.

**Table 1:** General characteristics (gender, age), treatment duration, underlying diseases and previous intensive care unit admission and antibiotic therapy history of the three cohorts

Characteristic/underlying disease	cefepime + vancomycin (Total = 20) n/(%)	ceftazidime + vancomycin (Total = 31) n/(%)	meropenem + vancomycin (Total = 119) n/(%)	p	Overall cohort (Total = 170) n/(%)
Female	8 (40%)	15 (48.4%)	69 (58%)	0.255	92 (54.1%)
Male	12 (60%)	16 (51.6%)	50 (42%)	0.255	78 (45.9%)
Age	49,35 ± 15.63	44,16 ± 14.96	49,13 ± 15.69	0.773	48.2 ± 15.6
Treatment duration	16,7 ± 8.38	11.9 ± 6.40	16,3 ± 10.3	0.066	15.6 ± 9.6
Intracranial tumor	6 (30)	12 (38.7%)	46 (38.7%)	0.754	64 (37.6%)
Intracranial hemorrhage	3 (15%)	1 (3.2%)	20 (16.8%)	0.152	24 (14.1%)
Hydrocephalus	7 (35%)	7 (22.6%)	18 (15.1%)	0.091	32 (18.8%)
Shunt	5 (25%)	5 (16.1%)	18 (15.1%)	0.544	28 (16.5%)
Lumbar drainage	1 (5%)	6 (19.4%)	13 (10.9%%)	0.261	20 (11.8%)
External ventricular drainage	3 (15%)	6 (19.4%)	27 (22.7%)	0.711	36 (21.2%)
Admission in intensive care unit during the previous one month	4 (20%)	8 (25.8%)	72 (60.5%)	0.000053	84 (49.4%)
Any antibiotherapy during the previous one month before the meningitis episode	5 (25%)	15 (48.4%)	74 (62.2%)	0.005773	94 (55.3%)

### Clinical presentation and diagnosis

Out of the total of 170 cases, 113 cases (66.5%) presented with fever, 92 cases (54.1%) exhibited disturbances in consciousness, 61 (36%) had neck stiffness, and 39 cases (22.9%) had all three symptoms, known as the classical triad. Additionally, 20 (11.8%) cases had convulsions and 55 (32.4%) had nausea and vomiting (Table 2).

**Table 2:** Clinical characteristics and CSF findings on the day empirical antibiotic was started, and meningitis etiology of the three cohorts

Finding	cefepime + vancomycin (Total = 20) n/(%)	ceftazidime + vancomycin (Total = 31) n/(%)	meropenem + vancomycin (Total = 119) n/(%)	p
Fever	13 (65%)	24 (77.4%)	76 (63.9%)	0.358
Disturbances in level of consciousness	8 (40%)	14 (45.2%)	70 (58.8%)	0.159
Glasgow coma score	13.65 ± 2.18	13.76 ± 2.62	11.85 ± 3.37	0.003
Neck stiffness	5 (25%)	8 (25.6%)	48 (40.3%)	0.180
Convulsions	3 (15%)	2 (6.5%)	15 (12.6%)	0.569
Nausea and vomiting	5 (25%)	10 (32.3%)	40 (33.6%)	0.748
Blood leukocyte (/mm^3^)	12,803 ± 5384	14,058 ± 6053	15,748 ± 8867	0.239
Blood leukocyte count(> 10.000/mm^3^)	12 (60%)	24 (77.4%)	92 (77.3%)	0.240
Blood CRP (mg/dl)	10.99 ± 12.31	14.75 ± 14.85	16.10 ± 16.79	0.455
CSF pleocytosis (mean, range)	745 ± 350 (170–1340)	1164 ± 1552 (80–8000)	1527 ± 3083 (10–20350)	0.441
CSF leukocyte count (mean, range/mm^3^)	144.0 ± 87.79 (62–348)	241.23 ± 287.27 (28–1210)	217.33 ± 181.90 (2–917)	0.474
CSF glucose (mean, range)	61.11 ± 30.8 (30–122)	38,70 ± 31.03 (2–112)	42,05 ± 29.73 (0–144)	0.160
Gram stain positive	1 (5%)	10 (32.3%)	31 (26%)	0.072
CSF culture negative	13 (65%)	11 (35.5%)	34 (28.6%)	0.006
*Acinetobacter baumannii*	0 (0%)	4 (12.9%)	20 (16.8%)	0.132
*Escherichia coli*	0 (0%)	0 (0%)	9 (75.7%)	0.130
*Klebsiella pneumoniae*	0 (0%)	2 (6.5%)	8 (67.2%)	0.491
*Pseudomonas aeruginosa*	0 (0%)	3 (9.7%)	6 (5%)	0.313
*Staphylococcus aureus*	2 (10%)	4 (12.9%)	13 (10.9%)	0.937
Coagulase-negative staphylococci	3 (15%)	4 (12.9%)	14 (11.8%)	0.915
Ceftazidime resistance among Gram-negative bacteria	0 (0%)	5 (45.4%)	37 (67.2%)	NA
Cefepime resistance among Gram-negative bacteria	0 (0%)	5 (45.4%)	28 (50.9%)	NA
Any methicillin-resistant staphylococci among staphylococci	4 (80%)	5 (62.5%)	19 (70.3%)	0.796
Any methicillin-sensitive staphylococci among staphylococci	1 (20%)	3 (37.5%)	8 (29.6%)	
Any carbapenem-sensitive-Gram-negative bacteria among Gram-negative bacteria	1 (100%)^a^	7 (63.6%)	33 (60%)	NA
Any carbapenem-resistant-Gram-negative bacteria among Gram-negative bacteria	0 (0%)	4 (36.3%)^b^	19 (34.5%)^c^	
*Enterococcus faecalis*	0 (0%)	1 (3.2%)	2 (1.7%)	0.648
*Enterococcus faecium*	0 (0%)	0 (0%)	2 (1.7%)	0.648
VRE	0 (0%)	0 (0%)	1 (0.8%)	0.806
Other pathogens	2 (10%)	3 (9.7%)	16 (13.4%)	0.803
Magnetic resonance imaging performed	13 (65%)	21 (67.8%)	98 (82.4%)	0.077
Magnetic resonance imaging no pathologic finding	0 (0%)	3 (9.7%)	6 (5%)	0.313
Ventriculitis in MRI	0 (0%)	1 (3.2%)	1 (0.8%)	0.333
Shunt removal	4 (20%)	3 (9.7%)	12 (10.1%)	0.410

^a^One case of cefepime and ceftazidime sensitive *Enterobacter aerogenes*

^b^one case carbapenem-resistant *K.pneumoniae*, three cases carbapenem –resistant *A. baumannii*

^c^One case carbapenem-resistant *K.pneumoniae*, one case carbapenem-resistant Citrobacter and 17 cases carbapenem –resistant *A. baumannii*

The mean levels for white blood cells and CRP were 15,089 ± 8106/mm^3^ and 15.20 ± 15.93 mg/dl, respectively. Out of 170 patients, 128 (75%) had leukocytosis (> 10,000/mm^3^). Additionally, 32 cases did not have leukocytosis but showed polymorphonuclear leukocyte predominance (> 64%) (Table 2).

The mean CSF leukocyte count was 1365 ± 2668/mm^3^ (range 10–20350/mm^3^), while the mean CSF protein and the mean CSF glucose levels were 215.15 ± 195.26 mg/dl (range 2–917 mg/dl) and 43.04 ± 30.21 mg/dl (range 0–144 mg/dl), respectively.

A total of 42 patients (24.7%) tested positive for Gram-stain, while 107 cases (62.9%) tested positive for CSF culture. No bacteriological agent was found in 63 cases (37.1%). Of the 66 bacteria isolated in CSF culture positive cases, 61.7% were Gram-negative while 46.7% were Gram-positive. The most common etiologic agent was *A. baumannii*, followed by coagulase-negative staphylococci and *S. aureus* (Table 2). Nine cases (5.3%) were found to have mixed infections. Twenty-two (91.7%) of the *A. baumannii* strains were resistant to carbapenem, and none were intermediately-resistant. Of all the Gram-negative bacteria, 9.2%, 64.6%, 52.3%, and 38.4% were ESBL producers, resistant to ceftazidime, cefepime, and carbapenem, respectively.

Although the rate of methicillin resistance among CNS strain was 71.4%, it was 63.1.% among *S. aureus* strains. Fortunately, there were no cases of fungal meningitis. However, one (0.6%) of the causative agents was vancomycin-resistant *E. faecium*.

Magnetic resonance imaging (MRI) was conducted on 77% of the overall cohort (132 patients). Out of the 132 patients, 10 cases (7.57%) showed normal MRI results. The most common MRI findings were postoperative changes (46.9%), hydrocephalus (28%), intracranial hemorrhage (9%), and abscess (6.1%). Besides, the MRI detected ventriculitis in two cases (1.5%).

According to Table 2, there was no significant difference among the three treatment groups in relation to symptoms, blood WBC, CRP, CSF bacterial etiology, CSF findings, MRI performed cases, cases with normal MRI and ventriculitis in MRI. However, the MV cohort had a significantly lower mean GCS score, and the CV cohort had a greater number of CSF culture-negative cases.

### Clinical efficacy

The clinical response on day 3–5 ranged between 45–54.8%. However, there was no significant difference between the three groups (*p* = 0.472, Table 3). A total of 56 cases that had positive CSF culture (32.9%) did not have a repeated CSF culture on day 3–5 or died before that time point. Nonetheless, there was no significant difference in day 3–5 microbiological responses between the three groups (*p* = 0.736, Table 3).

**Table 3:** Clinical outcomes, mortality, and sequalae of the three cohorts

Outcome	cefepime + vancomycin (Total = 20) n/(%)	ceftazidime + vancomycin (Total = 31) n/(%)	meropenem + vancomycin (Total = 119) n/(%)	p
Clinical response on day 3–5	9/20 (45%)	17/31 (54.8%)	61/119 (51.2%)	0.472
Microbiological response on day 3–5	7/12 (58%)	17/25 (68%)	32/54 (59%)	0.736
Needed change in antibiotic therapy after microbiological documentation	0/5 (0%)	4/20 (20%)	18/76 (23.7%)	0.344
End of treatment clinical success	16/20 (80%)	17/31 (54.8%)	69/119 (57.9%)	0.143
End of treatment clinical success in CSF culture negative cases	11/13 (85%)	8/11 (73%)	26/34 (76%)	0.762
End of treatment clinical success in CSF culture in carbapenem-sensitive Gram-negative bacteria	0/1 (0%)	5/6 (83.3%)	22/31 (70.9%)	NA
End of treatment clinical success in CSF culture in lumbar or ventricular rainage cases (ijncluding those with shunts at the time of diagnosis)	8/9 (88.9%)	10/17 (58.8%)	28/58 (48.3%)	0.069
End of treatment clinical success in carbapenem-resistant Gram-negative bacteria	0/0 (0%)	0/4 (0%)	0/18 (0%)	NA
End of treatment clinical success in methicillin-resistant staphylococci	4/4 (100%)	1/5 (20%)	14/19 (73.7%)	NA
End of treatment clinical success in ampicillin-resistant enterococci	0/0 (0%)	1/1 (100%)	0/4 (0%)	NA
Mortality due to any reason at EOT	2/20 (10%)	7/31 (22.5%)	23/119 (19.3%)	0.489
Infection related mortality at EOT	2/20 (10%)	5/31 (16.1%)	16/119 (13.4%)	0.821
Day 30 (after empirical treatment start) all cause mortality	3/20 (15%)	7/31 (22.6%)	31/119 (26%)	0.551
Day 30 (after empirical treatment start) all cause mortality in microbiologically confirmed cases	2/7 (28.6%)	6/20 (30%)	26/85 (30.6%)	0.993
Day 30 (after empirical treatment start) all cause mortality in microbiologically unconfirmed cases	1/13 (7.7%)	1/11 (9,1%)	5/34 (14.7%)	0.759
All cause mortality among cases with EOT success, 30 days follow up after EOT	1/16 (5.55%)	0/17 (0%)	12/69 (17.39%)	0.101
Overall clinical success	13/20 (65%)	16/31 (51.6%)	54/119 (45.3%)	0.252
Overall clinical success in any microbiologically unconfirmed meningitis	9/14 (64.2%)	6/11 (54.5%)	22/38 (57.8%)	0.874
Overall clinical success in any microbiologically confirmed meningitis	4/6 (66.6%)	10/20 (50%)	32/81 (39.5%)	0.337
Reinfection during one month follow up among cases with EOT success	0/16 (0%)	0/17 (0%)	2/69 (2.89%)	0.613
Relapse during one month follow up among cases with EOT success	0/16 (0%)	1/17 (5.88%)	1/69 (1.44%)	0.411
Any sequalae	0 (0%)	0 (0%)	33 (27.8%)	0.00015
Hematoma	0 (0%)	0 (0%)	2 (1.7%)	0.648
Hydrocephalus	0 (0%)	0 (0%)	3 (2.5%)	0.519
Abscess	0 (0%)	0 (0%)	7 (5.9%)	0.209
Disorder of cognitive functions	0 (0%)	0 (0%)	10 (8.4%)	0.102
Hemiparesis/hemiplegia	0 (0%)	0 (0%)	9 (7.6%)	0.152
Any adverse effect	1 (5%)	4 (12.9%)	6 (5%)	0.222
Antibiotic switch due to side effects	0 (0%)	1 (3.2%)	1 (0.8%)	0.478

The clinical success rate at the EOT was 80%, 54.8%, and 57.9% for the CV, CFV, and MV cohorts, respectively (*p* = 0.143, Table 3). There was no significant difference in EOT clinical success rates among the CSF culture-negative subgroups. Overall, vancomycin demonstrated an EOT clinical success rate of 60.6% (20/34) in the subgroup of methicillin-resistant staphylococci + ampicillin-resistant enterococci. Treatment failure occurred in two patients with vancomycin MIC > 1mg/l, but EOT success was achieved in 9 out of 10 patients with vancomycin MIC ≤ 1mg/l (*p* = 0.007). Nevertheless, as expected, the EOT clinical success rate was 0% in the carbapenem-resistant HCAM subgroup in the overall cohort (0/23).

Finally, there was no significant difference in terms of overall clinical success rate between the treatment groups (*p* = 0.252), as well as for both microbiologically confirmed (*p* = 0.874) and microbiologically unconfirmed meningitis subgroups (*p* = 0.337) (Table 3).

### Sequalae

The overall rate of sequela was 19.4%. Interestingly, no sequela was reported in the CV or CFV groups, while all sequelae were in the MV group, any sequalae was significantly higher than CF or CFV groups (*p* = 0.0015). However, there was no significant difference in terms of any sequela subgroup between the CV, CFV, and MV cohorts (see Table 3).

### Mortality

Overall ACM on day 30 of HCAM empirical treatment was 24.1% and did not differ significantly between the three groups as well as in möicrobioogically confirmed or unconfirmed subgroups (Table 3). At EOT (CV-CFV-MV), the overall mortality rate was 18.8%. The lowest mortality rate was observed in the CV cohort (10%), while the highest was observed in the CFV cohort (22.5%) (*p* = 0.489). The infection-related mortality rate during meningitis treatment (CV-CFV-MV) was 13.5%, with no significant difference among the three cohorts (*p* = 0.821). Thirteen cases died during the 30-day follow-up period for those with successful EOT (*p* = 0.101). Table 3 provides a summary of the mortality data.

### Adverse events

One participant in the CV cohort experienced an adverse event (AE) of abdominal pain. In the CFV cohort, four participants developed AEs, with two complaining of diarrhea, one reporting abdominal distention, and one requiring a drug switch due to an abnormal liver function test. Finally, six participants in the MV cohort experienced AEs, with three reporting nephrotoxicity, two experiencing a local reaction at the IV entrance site, and one requiring a drug switch due to an allergic reaction. The number of participants who experienced AEs that necessitated a change in therapy was similar across the three cohorts (*p* > 0.05, see Table 3).

## Discussion

HCAM is a very serious type of healthcare-associated infection that poses significant patient risks. The HCAM guidelines recommend ceftazidime, cefepime, or meropenem + vancomycin as empirical therapy options [5]. Although these combinations are the most commonly suggested regimens [1, 2, 4, 5], there has been no controlled or uncontrolled comparative analysis to determine the efficacy of these options. Cefepime has an advantage over ceftazidime in that it is resistant to inducible Ampc type beta-lactamases, while ceftazidime and cefepime both have the disadvantage of being susceptible to most ESBLs compared to meropenem. However, all three options are sensitive to carbapenemases [13, 14]. Using only one option for continuous antibiotic pressure may contribute to resistance; therefore, increasing the number of available empirical therapy options could increase the effective consumption periods of the available antibiotic options [13]. This study was conducted to determine whether these three options may be alternative to each other.

HCAM is a relatively rare nosocomial infection. However, in centers with overloaded neurosurgery clinics, it may comprise a more significant part of overall nosocomial infections. While HCAM is rare, it can lead to a mortality rate of 16–40.8% [15–21] and significant morbidity. In our cohort, overall mortality at EOT (CV-CFV-MV) was 18,8%. The lowest mortality was observed in CV cohort (10%) and highest in CFV cohort (22.5%) (*p* = 0.489) all of which are compatible with the results published in the literature [15–21]. It is worth noting that some patients experienced a relapse or developed other nosocomial infections, leading to additional mortality during the follow-up period. Hence, in our study, after 18.8% EOT mortality an additional 13 (7.6%) patients died until day 30 follow up. However, there was no significant difference in terms of mortality analysis between the three treatment groups.

As our study was a retrospective cohort study, it was not possible to balance the cohorts in all parameters including patient numbers or culture-negative cases in the three cohorts. MV combination was used more commonly than CV or CFV combination in the study centers, which created an imbalance in disfavor of CV and CFV cohorts. Additionally, the GCS at the start of empirical antibiotic therapy, the number of patients managed in the ICU, and the number of cases that received antibiotics in the previous month of the HCAM episode were significantly disfavored in the MV arm. It is possible that all these disadvantages, which are risk factors for ESBL, might have caused selection of meropenem rather than cefepime or ceftazidime. Furthermore, sequalae were more common in the MV arm, which may be due to the significantly lower GCS score and significantly higher ICU need in that cohort [1, 4]. However, the EOT clinical success, day-30 mortality, and overall clinical success at the end of the one-month follow-up did not differ significantly between the three cohorts.

Nosocomial *Acinetobacter* infections, including meningitis, are becoming increasingly common in ICUs. According to a recent systematic review of HCAM in Turkey [3], *Acinetobacter* spp. caused 30.7% of 899 CSF culture positive nosocomial meningitis episodes. The pooled carbapenem-resistance rate was 37.5%. In addition, carbapenem-resistant *Enterobacteriales* are increasingly being reported as a cause of HCAM [22, 23]. However, carbapenems, ceftazidime, and cefepime have the disadvantage of not covering carbapenem-resistant strains. As a result, all three treatment arms evaluated in our cohorts failed in the carbapenem-resistant subgroup.

Herein, we detected the overall clinical success rate with vancomycin as 60.6% in the ampicillin-resistant enterococci and methicillin-resistant staphylococci subgroup. As glycopeptides are relatively large molecules, their ability to penetrate the central nervous system is limited. Strains with a low MIC (< 1mg/l) are expected to have a higher likelihood of clinical success. Hence, we found that clinical success at EOT was lower in patients with a vancomycin MIC > 1mg/l compared to those with a MIC ≤ 1mg/l (*p* = 0.001) [9, 24, 25].

The reported penetration rates of the included antimicrobial agents into inflamed CSF are as follows: 20–40% for ceftazidime [26], 10.3% for cefepime [27], 39% for meropenem [27], and 30% for vancomycin [27]. However, the drug levels were not measured in any of the study centers.

Our study has several limitations i) its retrospective design ii) with a relatively small number of cases in the ceftazidime and cefepime groups, it was underpowered to demonstrate the efficacy of the three empirical treatment regimens in well-balanced cohorts and also unable to show the outcome differences in methicillin-resistant versus sensitive cases, as well as 3^rd^ generation and carbapenem resistant bacterial strains iii)the absence of antibiotic levels, pharmacokinetic and pharmacodynamic data including vancomycin, which were not measured in any of the cases iv) repeated lumbar puncture was not repeated In all cases vi) we did not analyse the neutrophil rates of CSF samples v) all the included cases fulfilled the inclusion criteria but we did not further examine the excluded cases) vi) Though we could include the meningitis episodes developping after 90 days we wanted to stay within the 90 days period in implant cases [28]. Herein, our primary objective was to compare the efficacies of empirical therapy and assess EOT clinical success and EOT mortality as the most critical outcomes. However, due to the fact that EOT not having a fixed time point, we conducted further analysis of day-30 ACM, which could have been impacted by revisions to intravenous/intrathecal antibiotic therapy after the etiology was elucidated. Nevertheless, needing such modifications was considered an unsuccessful outcome, and analysis of these modifications was kept outside the scope of this study. Additionally, infection related mortality was considered as per the treating physician. Due to the inability to perform autopsies, definitive reasons for mortality could not be determined. Our analysis indicated considerable mortality, relapse, and reinfection after the EOT, suggesting potential problems in infection control or reaching the best available therapy (especially in carbapenem-resistant Gram-negative cohort) at the study centers. Despite these limitations, to our knowledge, this is the first and the largest dataset evaluating the microbiological and clinical efficacy of empirical therapy regimens in HCAM, providing valuable insights into this area of study.

In conclusion this retrospective cohort study revealed no significant difference in EOT clinical success, EOT mortality, overall clinical success, and day-30 ACM among the three treatment arms. Our study was the first to compare three empirical therapy regimens in HCAM. While all three options had basicdisadvantages against carbapenem-resistant strains, vancomycin was found to have average efficacy against methicillin-resistant staphylococci. We suggest a randomized controlled trial to further analyze these three regimens. In addition, we suggest evaluating empirical therapy regimens that may be effective against carbapenem-resistant Gram-negative bacteria. We strongly recommend developing speedy diagnostic systems for HCAM, similar to those used in community-acquired meningitis or other infections [29, 30]. Finally the medical community should seek more effective measures for infection control in HCAM.

## Data Availability

The data that support the findings of this study are available from the corresponding author (Dr Deniz Akyol) upon reasonable request.

## References

[1] Bardak-Ozcem S, Sipahi OR (2014). An updated approach to healthcare-associated meningitis. Expert Rev Anti Infect Ther.

[2] Ippolito M, Giarratano A, Cortegiani A (2022). Healthcare-associated central nervous system infections. Curr Opin Anaesthesiol.

[3] Sipahi OR, Nazli Zeka A, Taşbakan M, Pullukçu H, Arda B, Yamazhan T, Sipahi H, Ulusoy S (2017). Pooled analysis of 899 nosocomial meningitis episodes from Turkey. Turk J Med Sci.

[4] Van de Beek D, Cabellos C, Dzupova O, Esposito S, Klein M, Kloek AT, Leib SL, Mourvillier B, Ostergaard C, Pagliano P, Pfister HW, Read RC, Sipahi OR, Brouwer MC, ESCMID Study Group for Infections of the Brain (ESGIB) (2016). ESCMID guideline: diagnosis and treatment of acute bacterial meningitis. Clin Microbiol Infect..

[5] Tunkel AR, Hasbun R, Bhimraj A, Byers K, Kaplan SL, Scheld WM, van de Beek D, Bleck TP, Garton HJL, Zunt JR (2017). 2017 Infectious Diseases Society of America's Clinical Practice Guidelines for Healthcare-Associated Ventriculitis and Meningitis. Clin Infect Dis.

[6] Tunkel AR, Hartman BJ, Kaplan SL, Kaufman BA, Roos KL, Scheld WM, Whitley RJ (2004). Practice guidelines for the management of bacterial meningitis. Clin Infect Dis.

[7] Van de Beek D, Drake JM, Tunkel AR (2010). Nosocomial bacterial meningitis. N Engl J Med.

[8] Karvouniaris M, Brotis A, Tsiakos K, Palli E, Koulenti D (2022). Current Perspectives on the Diagnosis and Management of Healthcare-Associated Ventriculitis and Meningitis. Infect Drug Resist.

[9] Sipahi OR, Bardak S, Turhan T, Arda B, Pullukcu H, Ruksen M, Aydemir S, Dalbasti T, Yurtseven T, Zileli M, Ulusoy S (2011). Linezolid in the treatment of methicillin-resistant staphylococcal post-neurosurgical meningitis: a series of 17 cases. Scand J Infect Dis.

[10] Garner JS, Jarvis WR, Emori TG, Horon TC, Hughes JM, Olmsted RN (1996). CDC definitions for nosocomial infections. APIC infection control and applied epidemiology: Principles and practice.

[11] Clinical and Laboratory Standards Institute (2013). Performance Standards for Antimicrobial Susceptibility Testing: Twenty-third Informational Supplement M100–S23.

[12] European Committee on Antimicrobial Susceptibility Testing Breakpoint tables for interpretation of MICs and zone diameters. Version 6.0, valid from 2016–01–01. Available from http://www.eucast.org/fileadmin/src/media/PDFs/EUCAST_files/Breakpoint_tables/v_6.0_Breakpoint_table.pdf. Accessed 12 Mar 2023.

[13] Sipahi OR (2008). Economics of antibiotic resistance. Expert Rev Anti Infect Ther.

[14] Rodríguez-Baño J, Gutiérrez-Gutiérrez B, Machuca I, Pascual A (2018). Treatment of Infections Caused by Extended-Spectrum-Beta-Lactamase-, AmpC-, and Carbapenemase-Producing *Enterobacteriaceae*. Clin Microbiol Rev.

[15] Durand ML, Calderwood SB, Weber DJ (1993). Acute bacterial meningitis in adults. A review of 493 episodes. N Engl J Med.

[16] Weisfelt M, Van de Beek D, Spanjaard L, de Gans J (2007). Nosocomial bacterial meningitis in adults. A prospective series of 50 cases. J Hosp Infect.

[17] Wang KW, Chang WN, Huang CR (2005). Post-neurosurgical nosocomial bacterial meningitis in adults: microbiology, clinical features, and outcomes. J Clin Neurosci.

[18] Reichert MC, Medeiros EA, Ferraz FA (2002). Hospital-acquired meningitis in patients undergoing craniotomy: incidence, evolution, and risk factors. Am J Infect Control.

[19] Dizbay M, Güzel Tunccan O, Arman D (2011). Factors associated with mortality in nosocomial central nervous system infections. ANKEM Derg.

[20] Erdem I, Hakan T, Metin F (2008). Clinical features, laboratory data management and the risk factors that affect the mortality in patients with postoperative meningitis. Neurol India.

[21] Van de Beek D, Brouwer MC, Thwaites GE, Tunkel AR (2012). Advances in treatment of bacterial meningitis. Lancet.

[22] Tuon FF, Rocha JL, Arend LN, Wallbach K, Zanin HA, Pilonetto M (2013). Treatment and outcome of nine cases of KPC-producing *Klebsiella pneumoniae* meningitis. J Infect.

[23] Guanghui Z, Jing L, Guojun Z, Hong L (2020). Epidemiology and risk factors of neurosurgical bacterial meningitis/encephalitis induced by carbapenem resistant Enterobacteriaceae. J Infect Chemother.

[24] Matsubara H, Makimoto A, Higa T (2003). Successful treatment of meningoencephalitis caused by methicillin-resistant *Staphylococcus aureus* with intrathecal vancomycin in an allogeneic peripheral blood stem cell transplant recipient. Bone Marrow Transplant.

[25] Arda B, Yamazhan T, Sipahi OR, İşlekel S, Büke Ç, Ulusoy S (2005). Meningitis due to methicillin-resistant *Staphylococcus aureus* (MRSA): review of 10 cases. Int J Antimicrob Agents.

[26] The Sanford Guide to Antimicrobial Therapy (2021). Dallas.

[27] Nau R, Sörgel F, Eiffert H. Penetration of drugs through the blood-cerebrospinal fluid/blood-brain barrier for treatment of central nervous system infections. Clin Microbiol Rev . 2010;23(4):858-83. 10.1128/CMR.00007-10 PMC2952976 20930076

[28] Sikora A, Zahra F. Nosocomial Infections. [Updated 2023 Apr 27]. In: StatPearls. Treasure Island (FL): StatPearls Publishing; 2023. Available from: https://www.ncbi.nlm.nih.gov/books/NBK559312/. Accessed 12 Mar 2023.

[29] Kahraman H, Tünger A, Şenol Ş, Gazi H, Avcı M, Örmen B, Türker N, Atalay S, Köse Ş, Ulusoy S, Işıkgöz Taşbakan M, Sipahi OR, Yamazhan T, Gülay Z, Alp Çavuş S, Pullukçu H (2017). Investigation of bacterial and viral etiology in community acquired central nervous system infections with molecular methods. Mikrobiyol Bul.

[30] Bastug A (2022). Rational use of syndromic approach. Mediterr J Infect Microb Antimicrob.

